# MiR-21: an environmental driver of malignant melanoma?

**DOI:** 10.1186/s12967-015-0570-5

**Published:** 2015-06-27

**Authors:** Bodo C Melnik

**Affiliations:** Department of Dermatology, Environmental Medicine and Health Theory, University of Osnabrück, Sedanstrasse 115, 49090 Osnabrück, Germany

**Keywords:** Environment, Exosome, Inflammation, Melanoma, Milk, MiR-21, Obesity, Pollution, UV-irradiation, Western lifestyle

## Abstract

Since the mid-1950’s, melanoma incidence has been rising steadily in industrialized Caucasian populations, thereby pointing to the pivotal involvement of environmental factors in melanomagenesis. Recent evidence underlines the crucial role of microRNA (miR) signaling in cancer initiation and progression. Increased miR-21 expression has been observed during the transition from a benign melanocytic lesion to malignant melanoma, exhibiting highest expression of miR-21. Notably, common BRAF and NRAS mutations in cutaneous melanoma are associated with increased miR-21 expression. MiR-21 is an oncomiR that affects critical target genes of malignant melanoma, resulting in sustained proliferation (PTEN, PI3K, Sprouty, PDCD4, FOXO1, TIPE2, p53, cyclin D1), evasion from apoptosis (FOXO1, FBXO11, APAF1, TIMP3, TIPE2), genetic instability (MSH2, FBXO11, hTERT), increased oxidative stress (FOXO1), angiogenesis (PTEN, HIF1α, TIMP3), invasion and metastasis (APAF1, PTEN, PDCD4, TIMP3). The purpose of this review is to provide translational evidence for major environmental and individual factors that increase the risk of melanoma, such as UV irradiation, chemical noxes, air pollution, smoking, chronic inflammation, Western nutrition, obesity, sedentary lifestyle and higher age, which are associated with increased miR-21 signaling. Exosomal miR-21 induced by extrinsic and intrinsic stimuli may be superimposed on mutation-induced miR-21 pathways of melanoma cells. Thus, oncogenic miR-21 signaling may be the converging point of intrinsic and extrinsic stimuli driving melanomagenesis. Future strategies of melanoma treatment and prevention should thus aim at reducing the burden of miR-21 signal transduction.

## Background

In developed countries, melanoma incidence has been rising with an annual increase between 3 and 7% for Caucasians since the mid-1950’s [1]. Thus, epidemiological data clearly point to the involvement of environmental factors in melanomagenesis. Lifestyle factors including occupational exposure, Western style nutrition, obesity, increased body mass index (BMI), recreational sun exposure, tanning and reduced physical activity may explain the relationship between environmental and socioeconomic factors and malignant cutaneous melanoma [2]. Recent data suggest that the common BRAF(V600E) mutation detected in melanoma is not associated with chronic sun exposure [3]. Thus, other environmental and epigenetic factors may play a role in melanomagenesis. Significant changes of microRNA (miR) expression in response to environmental exposure of humans have recently been reported [4]. MiRs are important posttranscriptional regulators controlling more than 30% of human mRNAs. Certain miRs such as miR-21 function as potent oncogenes [5] and play an important role in the initiation and progression of cancer [6]. OncomiRs affect all seven hallmarks of malignant cells: (1) self-sufficiency in growths signals, (2) insensitivity to anti-growth signals, (3) evasion from apoptosis, (4) limitless replicative potential, (5) angiogenesis, (6) invasion and metastasis, and (7) inflammation [6]. Current melanoma research focuses on the contribution of miR dysregulation in malignant melanoma [7–9] and its relation to BRAF and NRAS oncogenic mutations [10–12].

MiR-21 is highly expressed in melanoma cells and apparently plays a pivotal role in melanomagenesis [13]. A steady increase of miR-21 expression has been detected from benign to borderline melanocytic lesions and to primary cutaneous melanomas exhibiting an 8.6-fold overexpression of miR-21 [14]. UV-irradiation stimulates miR-21 expression in the skin [15]. Westernized nutrition, air pollution and inflammation all increase miR-21 signaling [4, 16, 17]. This review by means of translational research presents an analysis of the potential role of environmental and intrinsic factors that are associated with increased miR-21 expression and highlights miR-21-dependent pathways that may synergize in driving initiation and progression of malignant melanoma.

## MiR-21 and malignant melanoma

**Figure 1:**
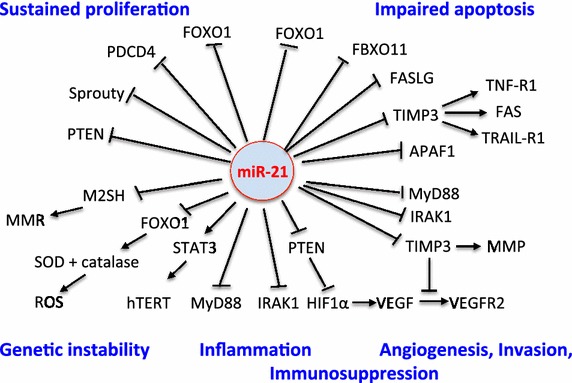
MiR-21 target mRNAs with potential impact on melanomagenesis. MiR-21 affects all major hallmarks of cancer: sustained proliferation, impaired apoptosis, genetic instability, angiogenesis and invasion, and inflammation (see list of abbreviations).

MiR-21 is a key oncogene, which is highly expressed in most cancers [18, 19]. Critical targets of miR-21 are mRNAs of tumor suppressor proteins, checkpoint regulators of cell cycle control, and intrinsic and extrinsic pathways of cellular apoptosis [20]. MiR-21 inhibits mRNA expression of crucial tumor suppressor proteins such as PTEN (phosphatase and tensin homolog) [21, 22], Sprouty1 and Sprouty2 [23–25], and PDCD4 (programmed cell death protein 4) [26–29]. MiR-21 is a negative regulator of p53 signaling [30] and stimulates the expression of the cell cycle promoter cyclin D1 [31]. MiR-21 induces tumor angiogenesis through targeting PTEN, leading to activated AKT and ERK1/2 signaling, thereby enhancing hypoxia-inducible factor 1α (HIF1α) and the expression of the vascular endothelial growth factor (VEGF) [32]. HIF1α is a crucial downstream target of miR-21 in regulating tumor angiogenesis [32–34]. Overexpression of HIF1α and HIF2α is linked to VEGF expression and poorer prognosis in malignant melanomas [35, 36] (Figure 1).

APAF1, apoptotic protease activating factor-1, which is the molecular core of the apoptosome, has been identified as a miR-21 target [37]. Metastatic melanomas often lose APAF1, a cell-death effector that acts with cytochrome c and caspase-9 to mediate p53-dependent apoptosis [38, 39]. There is an inverse correlation between APAF1 expression and melanoma progression [40, 41]. In fact, a significant difference in APAF1 mRNA expression between melanomas of Breslow thickness <1 and >4 mm has been detected [41]. Thus, there is good reason to believe that increased miR-21 expression via suppression of APAF1 prevents apoptosis in melanoma. Further studies confirm the involvement of miR-21 in the pathogenesis, carcinogenesis, progression and metastasis of malignant melanoma [13, 14, 42–45].

**Figure 2:**
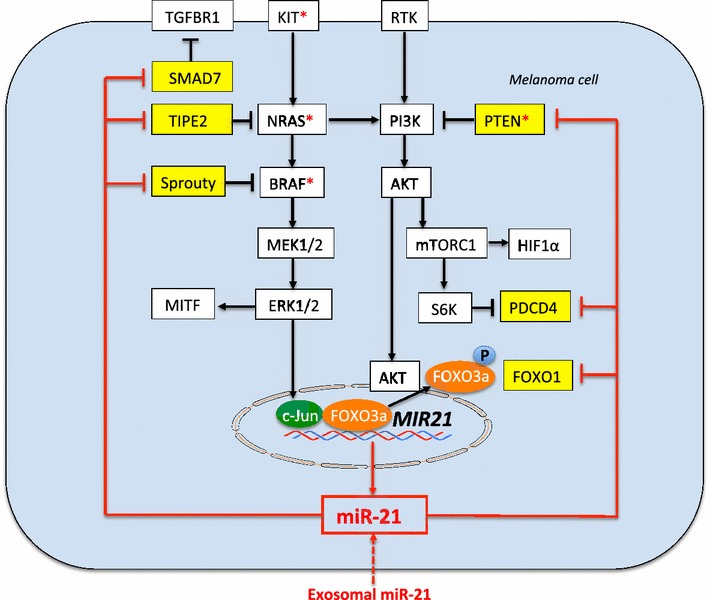
MiR-21 amplifies NRAS-BRAF-signaling by targeting Sprouty, enhances PI3K-AKT-mTORC1-signaling by targeting PTEN and PDCD4 enhances TGFβ signaling by suppression of SMAD7. Note: c-Jun is an activating transcription factor of *MIR21*, whereas FOXO3a suppresses *MIR21*; AKT-mediated phosphorylation of FOXO3a derepresses *MIR21*; miR-21 target mRNAs are labelled in *yellow*; *asterisk* indicates common mutations in malignant cutaneous melanoma.

Mutations in the BRAF gene and less frequently such as PTEN, KIT, CDK4, p53, MDM2, cyclin D1, AKT3, PI3Kα, or NRAS are involved in melanoma progression [10–12]. Important negative regulators of NRAS/BRAF/MEK/ERK- and the PI3K/AKT/mTORC1 signaling pathways are targets of miR-21 (Figure 2). Sprouty proteins are negative master regulators of RAF-RAS signaling [46]. MiR-21-mediated inhibition of Sprouty may thus promote mutated BRAF/NRAS signaling of melanoma cells. MiR-21 augments PI3K/AKT/mTORC1 signaling in cancer cells at various levels of the pathway [47, 48]. Recent evidence suggests that miR-21-mediated gene regulation interconnects with the AKT pathway [48]. In PC3 cells, miR-21 expression resulted in a dramatic increase in basal levels of the PI3K subunit p85 [49]. PI3K signaling is required for TGFβ-induced epithelial-mesenchymal (EMT)-like transition in human melanoma cells [50].

PTEN, which suppresses PI3K/AKT signaling, is often mutated in melanomas [12, 51]. Inactive mutated PTEN leads to overactivation of AKT, which inactivates FOXO tumor suppressor proteins [52].

Recent evidence shows that miR-21 not only targets PTEN, but also FOXO1 mRNA [53, 54]. Decreased FOXO activity has been associated with the malignant phenotype of melanoma cells [55, 56]. FOXO3a stimulates apoptosis by negatively targeting miR-21 [57]. MiR-21 suppresses the translation of pro-apoptotic FAS ligand (FASLG) [57]. It is noteworthy to mention that FoxO3a is a transcription factor promoting FOXO1 upregulation [58]. Thus, there exists a transcriptional and posttranslational regulatory network between FOXO3a, FOXO1 and miR-21 expression. Nuclear activity of FOXO transcription factors depends on PI3K/AKT-mediated phosphorylation of FOXO proteins [59]. As the miR-21 target PTEN counteracts the activity of PI3K, miR-21 has an upstream influence on nuclear FOXO-mediated transcription such as the expression of FOXO-dependent target genes like FASLG, cyclin D1, p21, superoxide dismutase and catalase involved in the regulation of apoptosis, cell cycle progression and defense against reactive oxygen species (ROS) [60]. Taken together, miR-21 enhances upstream and downstream PI3K/AKT signaling and reduces FOXO activities.

PDCD4 is a negative regulator of translation and acts as a tumor suppressor [61]. Notably, mTORC1 stimulates mRNA translation and protein synthesis via S6K1-mediated phosphorylation of PDCD4 (Figure 2) [62]. PDCD4 is suppressed in ~25% of human cell lines that are established from advanced melanoma lesions [63]. PDCD4 is a target of miR-21 [26–29]. Thus, overexpression miR-21 is involved in EMT by targeting PDCD4 and PTEN [64–67].

BTG2 (B-cell translocation gene 2) encodes an antiproliferative protein involved in the regulation of the G1/S transition of the cell cycle [68]. The tumor suppressor BTG2 is relevant to cell cycle control and cellular response to DNA damage [69]. BTG2 acts as a major downstream effector of p53-dependent proliferation arrest of mouse and human fibroblasts transduced with oncogenic RAS [70]. BTG2 is a known target of miR-21 [71]. Knockdown of miR-21 in B16 melanoma cells increases BTG2 levels [42], indicating that BTG2 is a miR-21 target in melanoma cells.

Another recently identified target of miR-21 is the insulin-like growth factor binding protein 3 (IGFBP3) [72]. Along with having a number of IGF-independent effects on cell growth and survival, IGFBP3 is known to modulate the activity of insulin-like growth factors (IGFs) [73]. In glioblastoma, miR-21-mediated downregulation of IGFBP3 expression promotes tumorigenesis [72]. Recent evidence underscores that IGFBP3 exerts a specific inhibitory effect on melanoma growth and dissemination [74].

Recently, the tumor suppressor FBXO11 has been identified as a novel miR-21 target [75]. FBXO11 is a component of the SKP1-CUL1-F-box ubiquitin ligase complex that targets proteins for ubiquitination and proteasomal degradation [76], a regulatory mechanism that plays a crucial role in the maintenance of genome stability [77]. FBXO11 promotes apoptosis by mediating the degradation of oncogenic BCL6. Notably, FBXO11 acts as a tumor suppressor in melanoma and has been shown to regulate apoptosis of B10BR mouse melanocytes [78].

Another recently detected miR-21 target is the tissue inhibitor of metalloproteinases 3 (TIMP3) [79]. Increased miR-21 expression enhances the invasive potential of melanoma cell lines through TIMP3 inhibition by (1) increasing matrix metalloproteinase activity [80], (2) by stimulating angiogenesis via TIMP3-mediated blockade of VEGF binding to VEGFR2 [80], and (3) by attenuating TIMP3-mediated apoptosis. Notably, TIMP3 exhibits potent antitumor activity in an animal model of melanoma [81] and induces apoptosis in melanoma cells [82]. Adenovirally expressed TIMP3 stabilzes tumor necrosis factor receptor-1 (TNF-R1), FAS, and TNF-related apoptosis, inducing ligand receptor-1 (TRAIL-R1) on melanoma cell surface, sensitizing these cells to apoptosis induced by TNF-α, anti-FAS-antibody and TRAIL [83]. Thus, the miR-21 target TIMP3 promotes apoptosis in melanoma cells by stabilizing three distinct death receptors and activating their apoptotic signaling cascade through caspase-8 (Figure 1).

Integrin-β4 (ITGβ4) is a novel miR-21 target gene and plays a role in the regulation of EMT, as it is remarkably derepressed after transient miR-21 silencing and downregulated after miR-21 overexpression. MiR-21-dependent changes of ITGβ4 expression significantly affect cell migration properties of colon cancer cells [84].

Expression of the L1 cell adhesion molecule (L1CAM) frequently occurs in human cancers and is associated with poor prognosis in cancers. It has recently been demonstrated that L1CAM induces the motility of B16F10 melanoma cells via the activation of MAPK pathways [85]. Notably, miR-21-3p has been identified as a positive regulator of L1CAM expression [86].

Overexpression of tropomyosin 1 (TPM1) in MCF-7 cells suppresses anchorage-independent growth, whereas overexpression of miR-21 increases tumor growth. Zhu et al. [87] concluded that miR-21 acts as an oncogene by suppressing TPM1. Indeed, increased expression of TPM1 has been associated with decreased invasive and motile activities of melanoma cells [88, 89].

## MiR-21 enhances genetic instability of melanoma cells

The DNA mismatch repair (MMR) protein MSH2 is an important tumor suppressor and crucial caretaker of the MMR, including transcription-coupled repair [90], homologous recombination [91], base excision repair [92], and plays an important role in mutation avoidance and microsatellite stability [93]. MSH2 is involved in the repair of UVA-induced oxidative DNA damage by base excision repair [94]. MSH2 gene mutations are present in the radial growth-phase of cutaneous malignant melanoma cell lines and can be further induced by UV-B [95]. Reduced or defective expression of MSH2 has been associated with high genomic instability, poor melanoma prognosis, and metastasis [95, 96]. Reduced expression or function of MSH2 is either a result of mutation-derived dysfunction of *MSH2* [94–97] or miR-21-mediated downregulation of MSH2 mRNA [98] (Figure 1).

Telomerase is reactivated in most cancers and there is accumulating evidence that this is a driver event in malignant melanoma [99–102]. hTERT is the catalytic subunit of telomerase, which regulates telomerase activity. Wang et al. [103] demonstrated in glioblastoma cells that enforced miR-21 expression increases hTERT expression mediated by STAT3, thereby promoting glioblastoma cell growth, whereas reduction of miR-21 represses hTERT expression in a STAT3-dependent fashion.

## Genetic and epigenetic changes in melanoma that upregulate miR-21

The development and progression of melanoma can be attributed to independent or combined genetic and epigenetic events [104]. The *MIR21* promoter region includes binding sites for activating protein 1 (AP-1) and signal transducer and activator of transcription 3 (STAT3) [19]. The AP-1 transcription factor c-Jun is highly expressed in melanoma cells [105]. AP-1/c-Jun activation results in enhanced expression of miR-21 [106–109]. Primary human melanocytes genetically modified to ectopically express BRAF(V600E) or NRAS (G12D) induce c-Jun expression [108–110] (Figure 2). RAS oncogenes are well-characterized inducers of AP-1 activity [109]. Indeed, oncogenic RAS induction in thyroid cells increases the expression of miR-21 [111]. Furthermore, an autoregulatory loop mediated by miR-21 and PDCD4 controls the AP-1 activity in RAS transformation [112].

Melanoma cells exhibit reduced expression of miR-125b, which is a negative regulator of c-Jun [113]. Thus, miR-125b/c-Jun/miR-21 signaling may represent a further pathway linked to miR-21-dependent melanomagenesis.

STAT3 is constitutively activated in a majority of human melanoma cell lines and tumor specimens [114, 115]. STAT3 activity is required for melanomagenesis and increases tumor invasiveness [116]. Noteworthy, STAT3 stimulates the expression of miR-21 [30, 40, 117]. In fact, STAT3/miR-21-signaling promotes proliferation and metastasis of B16 melanoma cells [42]. Inhibition of STAT3 is regarded as a potential therapeutic approach to target melanoma [118].

Approximately 50% of melanomas depend on mutant BRAF for proliferation, metastasis and survival [119]. Activation of STAT3 serine-727 and tyrosine-705 phosphorylations is promoted by BRAF(V600E) activity [118], whereas MEK inhibition decreases STAT3 phosphorylation in NRAS-mutant melanoma [119]. Furthermore, increased STAT3 signaling has been reported in primary oncogenic driver mutations of *KIT*, *ALK*, *ROS1*, *RET* and *NTRK1* [12]. In addition, RAS-induced expression of miR-21 is mediated through STAT3 signaling [20]. Taken together, common driver mutations of malignant melanoma via AP-1 and STAT3 activate miR-21 signal transduction.

MiR-182 is upregulated in melanoma cell lines [8, 120]. Aberrant miR-182 expression promotes melanoma metastasis by repressing FOXO3 and microphthalmia-associated transcription factor (MITF) [120]. It has recently been demonstrated that miR-182 is upregulated in melanoma cell lines after epigenetic modulation with the demethylating agents 5-aza-2′-deoxycytidine and trichostatin A [121]. It is concerning that miR-182 downregulates the expression of FOXO3 [121], which is a critical repressor of *MIR21* [57]. Thus, enhanced miR-182 signaling due to oncogenic mutations or epigenetic upregulation may synergistically augment miR-21 expression in melanoma. The intertwined connection between epigenetics and miRs such as miR-182 modulate the activity of the epigenetic machinery that plays a role in cancer development [122, 123].

Dermal fibroplasia appears to be related to the degree of atypia in dysplastic nevi [124]. Periadnexal fibrosis has been observed in melanoma in situ [125]. Thus, subepidermal fibroplasia may be a co-feature of melanomagenesis. Recent evidence links skin fibrosis to miR signaling [126]. MiR-21 promotes fibrogenic EMT of epicardial mesothelial cells involving PDCD4 and Sprouty-1 [127]. MiR-21 has been identified as central regulator of fibrosis [128].

Cancer-associated fibrosis plays an important role for the tumor stroma that supports cancer growth and invasion. Inhibition of miR-21 reduces liver fibrosis and prevents the development of hepatocellular carcinoma [129]. A paracrine signaling network involving PDGF-CC and PDGF receptor-α in a mouse model of malignant melanoma accelerates tumor growth through recruitment and activation of different subsets of cancer-associated fibroblasts (CAFs) [130]. TGF-β1 is a pivotal signal that promotes the generation of CAFs. Li et al. [131] demonstrated that miR-21 targets SMAD7, the upstream inhibitor of TGF-β1 signaling. MiR-21 binds to the 3′ UTR of SMAD7 mRNA and inhibits its translation [131]. Overexpression of miR-21 or the depletion of SMAD7 promoted CAF formation [131]. These observations point to an important contribution of miR-21 in shaping melanoma’s stromal microenvironment. Increased perilesional fibrosis may thus reflect the histologic result of enhanced miR-21 expression during the transition from a benign melanocytic lesion to malignant melanoma [14].

## Radiation-induced upregulation of miR-21

### Ultraviolet irradiation

Ultraviolet radiation (UV) is regarded as a major risk factor for melanoma development [132, 133]. Both, UV-B and UV-A are implicated in melanomagenesis [134]. UV-B irradiation of human HaCaT keratinocytes and a human squamous carcinoma cell line releases IL-6 [135, 136], which activates STAT3 signaling [137], and may consecutively increase the expression of miR-21 [30, 42]. Recent evidence underlines the predominant role of UV-A in melanomagenesis [138, 139]. Increased melanoma risk of airplane pilots has been linked to excessive UV-A exposure in the cockpit during flight time [140, 141]. In fact, miRs play an important role in photocarcinogenesis [142].

**Figure 3:**
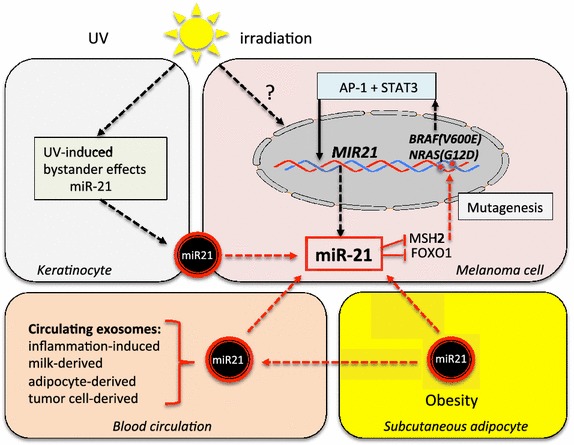
Working model of exogenous and endogeneous factors that increase exosomal miR-21 signaling in malignant melanoma. The common BRAF(V600E) and NRAS(G12D) mutations in cutaneous melanoma increase miR-21 expression by upregulating AP-1 and STAT3. Keratinocyte-derived exosomal miR-21 induced by UV-irradiation, exosomal miR-21 derived from the systemic circulation induced by dietary and environmental factors as well as obesity- and inflammation-associated exosomal miR-21 may enhance the total burden of miR-21 signaling of melanocytes promoting the transition to the malignant phenotype.

There is compelling evidence that miR-21 is upregulated by UV-A radiation of human skin [15]. Syed et al. [143] reported that both UV-A and UV-B irradiation of normal human epidermal keratinocytes activate STAT3. UV-B irradiation of the mouse epidermal JB6 cells induces the expression of miR-21 [144]. Radiation-induced bystander effects are established photobiological phenomena. Xu et al. [145] report significant upregulation of miR-21 in both directly irradiated cells and bystander cells. Notably, irradiated cells export miRs via exosomes [145–147] (Figure 3). Exosomes mediate cell–cell communication in a variety of biological processes and are considered as a new class of most important signalosomes that transport regulatory miRs between cells [148, 149]. Not only ionizing radiation but also UV-irradiation induces bystander effects in keratinocytes, fibroblasts and melanoma cells [150–153]. UV-induced expression of miR-21 in bystander keratinocytes may thus enhance exosomal miR-21 signaling, which affects gene regulation of adjacent melanocytic cells (Figure 3).

### Radiofrequency fields

There is a strong correlation between the accessibility to air travel and increasing melanoma incidence [154]. However, UV-irradiation may not represent the only electromagnetic spectrum associated with increased melanoma risk. A good correlation in time has been found for the rollout of FM/TV broadcasting networks and increasing melanoma prevalence [155]. Notably, enhanced STAT3 activation in response to 1,800 MHz radiofrequency fields has recently been demonstrated in astrocytes and microglia [156]. Compared to the general population, the incidence rate of gliomas is greater among melanoma patients [157]. In this regard, it is considerable that both glia cells and melanocytes derive from common neural crest cells [158]. Mutant gene expression of p53 in the peripheral zone of glioblastoma has been increased by 65% in patients using cell phones more than 3 h per day [159]. As in melanomas [13, 14], miR-21 is significantly overexpressed in glioblastomas [160]. The International Agency for Research on Cancer of the WHO classified radiofrequency electromagnetic fields as possibly carcinogenic to humans [161]. Thus, not only UV but also other electromagnetic spectra may activate oncogenic STAT3/miR-21 signaling.

## Upregulation miR-21 via circulating exosomes

### Milk exosome-derived miR-uptake

Bovine milk provides abundant bioactive exosomal miRs [162–164]. Exosomal miRs of pasteurized cow´s milk, such as miR-29b, are absorbed by humans in biologically meaningful amounts, which reaches the systemic circulation and affects gene expression of the milk consumer [16, 17]. It has been estimated that the 245 miRs detected in cow’s milk affect more than 11,000 human genes [16]. Milk has been proposed to represent an epigenetic transfection system that maintains maternal miR signaling to the newborn infant to promote postnatal growth [17]. Remarkably, bovine miRs of cow’s milk survive milk processing such as pasteurization, homogenization and refrigerated storage for over 2 weeks [165]. Due to lactase persistence, Caucasian populations are able to consume pasteurized fresh milk, which has been introduced into the food chain for daily consumption through the availability of widespread cooling technology since the mid-1950’s: The decade during which melanoma incidence increased substantially.

Exosomes facilitate miR transport over long distances [166]. Milk is obviously a mammalian interindividual miR-transfer system intended to provide maternal gene-regulatory signals to the newborn [17]. Intriguingly, bovine miR-21, a predominant miR constituent of cow’s milk [164, 167, 168], is identical to human miR-21 (http://www.mirbase.org). Notably, milk exosomes have been proven to be resistant against harsh degrading conditions [167, 168].

Recent epidemiological evidence links increased milk consumption to the pathogenesis of hepatocellular carcinoma [169]. In HepG2 hepatocellular carcinoma cells, interleukin 6 (IL-6) induces STAT3-dependent miR-21 transcription [170]. Michaëlsson et al. [171] reported a positive correlation between milk intake and overall mortality and increased IL-6 serum levels in two Swedish cohorts. Notably, increased serum IL-6 levels have been correlated with a worse prognosis of melanoma [172, 173]. Milk also delivers exosomal miR-155 [164], which is involved in STAT3-mediated tumorigenesis [174, 175]. MiR-155 enhances STAT3 expression by reducing suppressor of cytokine signaling 1 (SOCS1), a target of miR-155 [175, 176]. Grignol et al. [16] observed increased expression of both miR-21 and miR-155 during the progression of melanocytic lesions. Pieters et al. showed that commercial milk exosomes transmit TGF-β [168]. TGFβ signaling promotes a rapid increase in the expression of mature miR-21 through promoting the processing of primary transcripts of miR-21 (pri-miR-21) into precursor miR-21 (pre-miR-21) by the DROSHA complex [177]. Thus, intake of pasteurized milk may increase miR-21 signaling either through direct exosomal transfer of miR-21 or via STAT3- and TGFβ-mediated upregulation of miR-21 expression.

### Tumor exosome-derived miR-21

Not only milk provides miR-21 via exosome transfer but also tumor cells. Intriguingly, miR content of circulating tumor-derived exosomes has been found to correlate with the miR profile of the tumor [178, 179]. In patients with ovarian cancer, lung cancer and glioblastoma, miR-21 is one of the most abundant miRs detected in the patients’ circulating exosomes [178, 179]. The uptake of miR-21 delivering exosomes is facilitated via clathrin-mediated endocytosis and macropinocytosis [180]. Notably, it has been demonstrated that tumor cell-derived exosomes regulate target gene expression in normal cells [180]. MiR-21 is also one of the major exosomal miRs released from melanoma cells [44, 181, 182]. Indeed, plasma miR-21 levels have been associated with the tumor burden in cutaneous melanoma [44]. Thus, exosomal melanoma-derived miR-21 may promote melanoma invasion and distant metastasis. According to a recent concept, tumor-derived exosomal miRs such miR-21 may be involved in tumor-mediated immunosuppression [183]. For instance, nasopharyngeal cancer-derived miR-21 induces IL-10 and B-cells that suppress CD8+ T-cell activities [184]. Moreover, miR-21 is involved in the generation of myeloid-derived suppressor cells (MDSCs), which exert potent immunosuppressive activities [185]. Recent evidence underlines that high levels of MDSCs are associated with a poor overall survival of melanoma patients [186]. Furthermore, miR-21 targets two important regulatory checkpoints in the TLR signaling pathway, myeloid differentiation factor 88 (MyD88) and interleukin-1 receptor-associated kinase 1 (IRAK1) [187]. Moreover, tumor-secreted miR-21 can function via binding as ligands to murine TLR7 and human TLR8 in immune cells, thereby triggering a TLR-mediated prometastatic inflammatory response that ultimately may lead to tumor growth and metastasis [188].

## Age- and inflammation-induced miR-21

MiR-21 is a major miR component of human plasma that increases in association with age, inflammation, cardiovascular disease, and obesity [189–191]. STAT3 activation of miR-21 has been proposed as a mechanistic link between inflammation and cancer development [192]. Inflammasome activation and increased IL-1β signaling has recently been proposed to play a potential role in driving melanomagenesis [193]. Serum IL-1β is significantly increased in advanced melanoma patients [194]. Remarkably, it has been demonstrated in human aortic epithelial cells that IL-1β treatment induces a three to fourfold response of miR-21-3p expression compared with control treatment [195]. This can be explained by IL-1β-induced IL-6/STAT3 signaling [196].

Inflammation is a newly recognised hallmark of cancer that substantially contributes to the development and progression of malignancies [197]. Increasing evidence underlines the role that local immune response and systemic inflammation have in the progression of tumors and survival of cancer patients. It has recently been proposed that miR-21 plays a key role in the induction and resolution of inflammatory responses [198] and in the regulation of immune homeostasis [196]. Tumor necrosis factor-α-induced protein 8-like 2 (TIPE2), which belongs to the TNF-α-induced protein-8 family, is a negative regulator of innate and adaptive immunity and is a direct target of miR-21 [199, 200]. TIPE2 negatively regulates inflammation by switching arginine metabolism from nitric oxide synthase to arginase, which converts arginine to ornithine and urea [201]. Melanomas are auxotrophic for arginine due to the reduced expression of argininosuccinate synthetase-1 (ASS1), which is the rate-limiting enzyme for arginine biosynthesis [202]. Furthermore, miR-21-regulated TIPE2 controls innate immunity to RNA by targeting the PI3K-RAC pathway [203]. TIPE2 is able to suppress TNF-α-induced hepatocellular carcinoma metastasis by inhibiting ERK1/2 and NF-κB activation [204]. Downregulated TIPE2 is associated with increased cell proliferation and poor prognosis in non-small lung cancer [205]. Furthermore, TIPE2 provides a molecular bridge from inflammation to cancer by targeting the RAS signaling pathway [206, 207]. TIPE2 binds the RAS-interacting domain of the RALGDS (RAL guanine nucleotide dissociation stimulator) family of proteins, which are essential effectors of activated RAS. This binding prevents RAS from forming an active complex, thereby inhibiting the activation of the downstream signaling molecules RAL and AKT [207]. Thus, miR-21 reduces TIPE2-mediated suppression of RAS-induced tumorigenesis, which may have a potential impact on RAS-driven melanomagenesis.

## Association of nutrition and lifestyle factors overexpressing miR-21

### High glucose consumption

Western diet is characterized by excessive consumption of sugar and hyperglycemic carbohydrates resulting in excessive glucose intake. About 75% of all foods and beverages contain added sugar in a large array of forms. Consumption of soft drinks has increased fivefold since 1950 [208]. It has recently been demonstrated that miR-21 is upregulated in a time-dependent manner in response to high concentration glucose stimulation in Raw 264.7 macrophages [209]. Inhibition of miR-21 increases mRNA and protein levels of PDCD4 [209]. Thus, the steady increase in daily glucose consumption may be related to the total burden of miR-21.

### High fat intake and obesity

Several studies demonstrated an association between increased BMI and melanoma incidence and mortality [3]. MiR-21 is involved in adipocyte differentiation and is upregulated in subcutaneous adipose tissue of obese individuals [210, 211]. A long-term high-fat diet (HFD) upregulates murine miR-21 and induces obesity in mice [212]. MiR-21 is robustly expressed in human adipose tissue and positively correlates with BMI [211], whereas long-term inhibition of miR-21 reduces obesity in db/db mice [213]. Pandey et al. [214] provided evidence that HFD-induced obesity leads to increased melanoma progression in male C57BL/6J mice associated with enhanced Cav-1 and FASN expression in tumors from HFD mice. Cav-1 and FASN are coordinately regulated and Cav-1 interacts with FASN in melanoma cells [214]. In accordance with this, Malvi et al. [215] demonstrated that reduction of caloric intake by orlistat treatment of obese mice significantly diminishes melanoma progression. Interestingly enough, adipocytes secrete exosomes containing abundant miRs [216, 217]. In the B16F10 melanoma allograft model, adipose tissue conditioned media from HFD-induced obese mice increase lymphangiogenesis and lymph node metastasis [218]. Exposure to media from adipocyte cultures increases cell proliferation and reduces sensitivity of melanoma cells to apoptosis induced by cisplatin and docetaxel [219]. Notably, miR-21 has been shown to decrease chemosensitivity of cancer cells to cisplatin [220, 221] and docetaxel [222, 223]. Future studies should elucidate whether adipocyte-derived miR-21 may play a role in melanomagenesis (Figure 3).

### Alcohol consumption

There is epidemiological evidence that drinking alcohol is associated with an increased risk of melanoma [224, 225]. Expression of miR-21 and several of their target genes are regulated by acute psychological stress and have been correlated with alcohol consumption in a laboratory setting [226].

### Sedentary lifestyle

Sedentary lifestyle with insufficient physical activity is not only a risk factor for obesity but may be related to the increasing prevalence of melanoma in industrialized societies. In fact, US men and women exercising five to seven days per week have been reported to be at decreased risk of melanoma [227]. In accordance with this, a melanoma-protective effect of increased physical exercise has been reported in Greece [228]. Nielsen et al. [229] observed a decrease of circulating plasma miR-21 in response to chronic exercise. Thus, a sedentary lifestyle may adversely affect miR-21 signaling.

## Pollution-mediated upregulation of miR-21

Smoking and nicotine exposure upregulates the expression of miR-21 [67, 230]. Chronic arsenic exposure acts as a co-carcinogen in melanoma [231]. Arsenic exposure induces the expression of miR-21 [65, 232–235]. A recent study shows that miR-21 is involved in exosome-mediated intercellular communication between neoplastic and normal human bronchial epithelial (HBE) cells [236]. Exosomes derived from arsenite-transformed HBE cells stimulate proliferation of normal HBE cells, whereas exosomes from miR-21-depleted cells fail to stimulate proliferation. Collectively, these data support the concept that exosomal miR-21 is involved in cell–cell communication during carcinogenesis induced by environmental chemicals. Furthermore, air pollution and oxidative stress induce the expression of miR-21. It has been demonstrated that metal-rich particulate matter increases the expression of miR-21 in peripheral blood leukocytes [237]. Notably, miR-21 expression has been associated with exposure to diesel exhaust linked to increased plasma levels of 8-hydroxy-deoxyguanosine (8-OHdG) [238]. 8-OHdG is induced in DNA by oxidative stress and UV irradiation [239]. Indeed, melanoma patients with low expression of nuclear 8-OHdG have significantly longer survival times compared to those with high expression [240]. Thus, air pollution and urbanisation may affect melanomagenesis via environmental stressors that upregulate miR-21 expression (Table 1).

**Table 1:** Environmental and lifestyle factors suggested to upregulate miR-21 signaling

MiR-21 stimulus	Mode of action [Refs.]
UV-irradiation	IL-6, STAT3; exosome release [15, 135–137]
Cell phone use	STAT3 upregulation [156]
Smoking	Increased miR-21 expression [67, 230]
Alcohol abuse	Increased miR-21 expression [226]
Pollution	Increased miR-21 expression by particulate matter [237]
	Increased miR-21 expression by diesel exhaust [238]
Arsenic	Increased miR-21 expression [65, 232–235]
	Exosome release of bronchial epithelial cells [236]
Inflammation	IL-1β, IL-6, STAT3 [192–198]
Milk consumption	Uptake of milk-delivered exosomal miR-21 [16, 17, 162–168]
	Transfer of bovine miR-155, STAT3; IL-6 [169, 170, 173]
Obesity	Increased adipocyte miR-21 expression [210, 212]
	Adipocyte-derived exosome release [211–216]
Tumor diseases	Release of tumor cell-derived exosomes [44, 178–186]
Sedentary lifestyle	Physical exercise decreases miR-21 expression [229]
Higher age	Increased plasma miR-21 levels [189]

## Conclusions

Overexpression miR-21 is a common molecular feature of malignant melanoma [13]. MiR-21 plays a crucial role in regulatory circuits involving epigenetic switches required for the transformation of cancer cells [192]. MiR-21 expression is upregulated by environmental, epigenetic and genetic changes that may all promote melanomagenesis. In comparison to epidermal keratinocytes, environment insults are apparently more critical for melanocytes, as these cells are considerably more resistant to apoptosis [241–243]. Thus, environmental and epigenetic factors that persistently increase miR-21 signaling may have predominantly long-lasting impacts on melanocytes. In this regard, melanoma appears to represent a prototype of a cancer that is promoted by environmental factors.

Apparently, the expression of miR-21 steadily increases during the transition of a benign melanocytic to a borderline and malignant lesion [14]. MiR-21 is an oncomiR that intersects with all hallmarks of cancer: (1) sustaining proliferative signaling, (2) evading growth suppressors, (3) activating invasion and metastasis, (4) enabling replicative immortality, (5) inducing angiogenesis, (6) resisting cell death, and (7) inflammation [197, 244] (Figure 1). Exosome-derived miRs have been implicated to play a major role in the development and progression of cancer by epigenetic reprogramming [245]. Westernized environments, nutrition, and lifestyle may modify epigenetic programming via miR-21.

The most common BRAF and RAS mutations of melanoma are associated with increased miR-21 expression (Figure 2). Upregulated miR-21 expression induced by various lifestyle factors and environmental conditions of industrialized countries may be the converging point of oncogenic stimuli promoting melanomagenesis. Increased expression of miR-21 has been observed in association with UV-irradiation and other electromagnetic radiation, smoking, pollution with exposure to particulate matter and diesel exhaust. MiR-21 signaling of melanoma cells may be upregulated by exosomal transfer of miR-21.

Exosomes have been identified as major players maintaining a molecular crosstalk between tumor cells and cell of the innate immune system [246]. Exosomes may reach pigmented lesions either via bystander effects of UV-irradiated keratinocytes or via the circulation or underlying subcutaneous adipose tissue in obesity. Thus, environmental and intrinsic factors may work in an additive or synergistic manner, thereby increasing the total individual burden of miR-21 signaling (Figure 3). It is of special concern that miR-21 not only promotes melanoma progression, but that it is also involved in the initiation of melanoma. MiR-21-mediated suppression of MSH2-dependent DNA mismatch repair, insufficient FoxO-controlled ROS-homeostasis, imbalances of FBXO11-regulated proteasomal degradation of critical proteins involved in cell proliferation and apoptosis, and miR-21-stimulated telomerase activity may all increase genetic instability promoting the risk of mutagenesis. In this regard, miR-21 may represent the common denominator of accumulating environmental and intrinsic stressors that drive the initiation and progression of malignant melanoma (Figure 3).

The versatility of miRs as molecular tools inspires the design of novel strategies for the treatment of malignant melanoma [247]. As suggested for glioblastoma and ovarian carcinoma [248, 249], anti-miR-21 treatment may be a promising option for the treatment of malignant melanoma [79]. It has been demonstrated that the curcumin analog EF24 exhibited potent anticancer activity in B16 murine melanoma cells associated with a downregulation of miR-21 [250]. Furthermore, inhibition of miR-21 increases chemosensitivity in a variety of tumors [220–223]. There is further evidence that anti-miR-21 treatment downregulates the anti-apoptotic mitochondrial membrane protein BCL2 (B-cell leukemia 2), which blocks apoptotic cell death [251]. BCL2 is a MITF target gene involved in melanocyte and melanoma cell proliferation and survival [252, 253]. Combined inhibition of NF-κB and BCL2 triggers synergistic reduction of viability and induces apoptosis in melanoma cells [254]. Remarkably, miR-21 inhibition suppresses proliferation and migration of nasopharyngeal carcinoma and breast cancer cells through downregulation of anti-apoptotic BCL2 [255, 256]. Furthermore, disruption of miR-21 by transcription activator-like effector nucleases (TALENs) in cancerous cells lead to diminished cell transformation and increased expression of cell-environment interaction genes [257].

Taken together, melanoma is a model cancer system not only involving genetic but also environmental components [258]. At the molecular level, miR-21 links environmental exposure to melanomagenesis. Circulating exosomal miRs, especially miR-21, represents a very important signaling system of cell communication [259] that apparently mediates the impact of environmental and epigenetic factors in melanomagenesis. Circulating and locally generating exosomal miR-21 through various environmental stimuli may significantly contribute to the multistep process of melanomagenesis. Decreasing the input and magnitude of extrinsic and intrinsic stimuli that promote overexpression and release of exosomal miR-21 may thus be a very promising approach in the prevention and treatment of melanoma, the most serious human skin disease that is apparently promoted by common Western lifestyle factors.

## Abbreviations

AP-1: activating protein 1
APAF1: apoptotic protease activating factor 1
BCL2: B-cell leukemia 2
BMI: body mass index
BRAF: V-RAF murine sarcoma viral oncogene homolog B1
BTG2: B-cell translocation gene 2
CAF: cancer-associated fibroblast
EMT: epithelial-mesenchymal transition
FAS: FAS cell surface death receptor
FASLG: FAS ligand
FBXO11: F-box only protein 11
FOXO1: forkhead box O1
HIF-1α: hypoxia-inducible factor 1α
hTERT: human telomerase reverse transcriptase
MiR: micro-ribonucleic acid
IGFBP3: insulin like growth factor binding protein 3
IRAK1: interleukin 1 receptor-associated kinase 1
L1CAM: L1 cell adhesion molecule
MDSC: myeloid-derived suppressor cell
MITF: microphthalmia-associated transcription factor
MMR: DNA mismatch repair
MSH2: MutS, E. coli, homolog of, 2
mTORC1: mechanistic target of rapamycin complex 1
MyD88: nyeloid differentiation primary response gene 88
NRAS: neuroblastoma RAS viral oncogene homolog
8-OhdG: 8-hydroxydeoxyguanosine
PDCD4: programmed cell death protein
PI3K: phosphoinositol-3 kinase
PTEN: phosphatase and tensin homolog
RALGDS: RAL guanine nucleotide dissociation stimulator
ROS: reactive oxygen species
RTK: receptor tyrosine kinase
SOCS1: suppressor of cytokine signaling 1
STAT3: signal transducer and activator of transcription 3
TGFBR1: transforming growth factor beta receptor type 1
TIMP3: tissue inhibitor of metalloproteinase 3
TGFβ: transforming growth factor beta
TIPE2: tumor necrosis factor-alpha-induced protein 8-like 2
TNF-R1: tumor necrosis factor receptor-1
TPM1: tropomyosin 1
TRAIL-R1: TNF-related apoptosis inducing ligand receptor-1
UV: ultraviolet radiation
VEGF: vascular endothelial growth factor
